# Supplementary material for: ProteinShader: illustrative rendering of macromolecules
Source: BMC Struct Biol. 2009 Mar 30;9:19. doi: 10.1186/1472-6807-9-19 (PMC2672931; doi:10.1186/1472-6807-9-19)
Supplement: Additional file 1 — ProteinShader program without source code. This compressed file contains the complete ProteinShader program including associated libraries, but no source code. A README.txt file gives an overview of the ProteinShader distribution, and the index.html file in the help subdirectory has directions on getting started with the program as well as a set of tutorials. [file 1472-6807-9-19-S1.zip › ProteinShader-beta-0_9_4-binary/help/api/serialized-form.html]

Serialized Form (ProteinShader API)


|  |  |  |
| --- | --- | --- |
| **Overview** \| Package \| Class \| Use \| **Tree** \| **Deprecated** \| **Index** \| **Help** | |  |
| PREV   NEXT | **FRAMES**    **NO FRAMES**     **All Classes** |  |


---


# Serialized Form


---

**Package** **org.proteinshader.graphics.exceptions**

**Class org.proteinshader.graphics.exceptions.GlyphConfigException extends GlyphException implements Serializable**

**Serialized Fields**

### m\_configFilename

```
String m_configFilename
```

**Class org.proteinshader.graphics.exceptions.GlyphException extends Exception implements Serializable**

**Class org.proteinshader.graphics.exceptions.GlyphImageException extends GlyphException implements Serializable**

**Serialized Fields**

### m\_name

```
String m_name
```

---

### m\_x1

```
int m_x1
```

---

### m\_y1

```
int m_y1
```

---

### m\_x2

```
int m_x2
```

---

### m\_y2

```
int m_y2
```

---

### m\_bytesPerPixel

```
int m_bytesPerPixel
```

---

### m\_glyphWidth

```
int m_glyphWidth
```

---

### m\_glyphHeight

```
int m_glyphHeight
```

---

### m\_glyphSetImageWidth

```
int m_glyphSetImageWidth
```

---

### m\_glyphSetImageHeight

```
int m_glyphSetImageHeight
```

**Class org.proteinshader.graphics.exceptions.ShaderException extends Exception implements Serializable**

**Class org.proteinshader.graphics.exceptions.TextureException extends Exception implements Serializable**

---

**Package** **org.proteinshader.gui**

**Class org.proteinshader.gui.ProteinShaderGUI extends JFrame implements Serializable**

**Serialized Fields**

### m\_canvas

```
GLCanvas m_canvas
```

---

### m\_mediator

```
Mediator m_mediator
```

---

### m\_renderer

```
Renderer m_renderer
```

---

### m\_factory

```
WindowListenerFactory m_factory
```

---

**Package** **org.proteinshader.gui.components**

**Class org.proteinshader.gui.components.SpringLoadedJFrame extends JFrame implements Serializable**

**Serialized Fields**

### m\_parent

```
JFrame m_parent
```

---

### m\_position

```
OrientationEnum m_position
```

---

### m\_widthFactor

```
double m_widthFactor
```

---

### m\_heightFactor

```
double m_heightFactor
```

---

### m\_hideWhileParentInvisible

```
boolean m_hideWhileParentInvisible
```

---

### m\_hideWhileParentIconfied

```
boolean m_hideWhileParentIconfied
```

---

### m\_frameOpening

```
boolean m_frameOpening
```

---

### m\_timer

```
Timer m_timer
```

---

### m\_fractionVisible

```
double m_fractionVisible
```

---

**Package** **org.proteinshader.gui.components.controlpanel**

**Class org.proteinshader.gui.components.controlpanel.AntialiasingPanel extends JPanel implements Serializable**

**Serialized Fields**

### m\_factory

```
AntialiasingPanelListenerFactory m_factory
```

---

### m\_mediator

```
Mediator m_mediator
```

---

### m\_dialogOwner

```
Frame m_dialogOwner
```

---

### m\_sceneAntialiasBox

```
JCheckBox m_sceneAntialiasBox
```

---

### m\_halftoningAntialiasBox

```
JCheckBox m_halftoningAntialiasBox
```

---

### m\_jitterMenu

```
JComboBox m_jitterMenu
```

---

### m\_debug

```
boolean m_debug
```

**Class org.proteinshader.gui.components.controlpanel.AtomColorPanel extends ColorPanel implements Serializable**

**Serialized Fields**

### m\_atomModifier

```
AtomModifier m_atomModifier
```

**Class org.proteinshader.gui.components.controlpanel.AtomScalePanel extends JPanel implements Serializable**

**Serialized Fields**

### m\_factory

```
ScalePanelListenerFactory m_factory
```

---

### m\_mediator

```
Mediator m_mediator
```

---

### m\_dialogOwner

```
Frame m_dialogOwner
```

---

### m\_atomModifier

```
AtomModifier m_atomModifier
```

---

### m\_radioPanel

```
RadioPanel m_radioPanel
```

---

### m\_style

```
StyleEnum m_style
```

---

### m\_scaleSpinner

```
JSpinner m_scaleSpinner
```

---

### m\_applyButton

```
JButton m_applyButton
```

---

### m\_defaultButton

```
JButton m_defaultButton
```

**Class org.proteinshader.gui.components.controlpanel.AtomVisibilityPanel extends VisibilityPanel implements Serializable**

**Serialized Fields**

### m\_atomModifier

```
AtomModifier m_atomModifier
```

---

### m\_bondModifier

```
BondModifier m_bondModifier
```

**Class org.proteinshader.gui.components.controlpanel.CartoonColorPanel extends ColorPanel implements Serializable**

**Serialized Fields**

### m\_segmentModifier

```
SegmentModifier m_segmentModifier
```

**Class org.proteinshader.gui.components.controlpanel.CartoonSideChainsPanel extends JPanel implements Serializable**

**Serialized Fields**

### m\_factory

```
CartoonSideChainsPanelListenerFactory m_factory
```

---

### m\_segmentModifier

```
SegmentModifier m_segmentModifier
```

---

### m\_mediator

```
Mediator m_mediator
```

---

### m\_dialogOwner

```
Frame m_dialogOwner
```

---

### m\_radioPanel

```
RadioPanel m_radioPanel
```

---

### m\_alphaCarbonCheckBox

```
JCheckBox m_alphaCarbonCheckBox
```

---

### m\_spaceFillingButton

```
JButton m_spaceFillingButton
```

---

### m\_ballsAndSticksButton

```
JButton m_ballsAndSticksButton
```

---

### m\_sticksButton

```
JButton m_sticksButton
```

---

### m\_noneButton

```
JButton m_noneButton
```

**Class org.proteinshader.gui.components.controlpanel.CartoonVisibilityPanel extends VisibilityPanel implements Serializable**

**Serialized Fields**

### m\_segmentModifier

```
SegmentModifier m_segmentModifier
```

**Class org.proteinshader.gui.components.controlpanel.ColorPanel extends JPanel implements Serializable**

**Serialized Fields**

### m\_factory

```
ColorPanelListenerFactory m_factory
```

---

### m\_mediator

```
Mediator m_mediator
```

---

### m\_dialogOwner

```
Frame m_dialogOwner
```

---

### m\_radioPanel

```
RadioPanel m_radioPanel
```

---

### m\_chooserButton

```
JButton m_chooserButton
```

---

### m\_default1Button

```
JButton m_default1Button
```

---

### m\_default2Button

```
JButton m_default2Button
```

---

### m\_chooser

```
JColorChooser m_chooser
```

---

### m\_chooserDialog

```
JDialog m_chooserDialog
```

**Class org.proteinshader.gui.components.controlpanel.ControlPanel extends JPanel implements Serializable**

**Serialized Fields**

### m\_selectorPanel

```
SelectorPanel m_selectorPanel
```

---

### m\_modifierPanel

```
ModifierPanel m_modifierPanel
```

**Class org.proteinshader.gui.components.controlpanel.DecorationsPanel extends JPanel implements Serializable**

**Serialized Fields**

### m\_factory

```
DecorationsPanelListenerFactory m_factory
```

---

### m\_mediator

```
Mediator m_mediator
```

---

### m\_dialogOwner

```
Frame m_dialogOwner
```

---

### m\_segmentModifier

```
SegmentModifier m_segmentModifier
```

---

### m\_radioPanel

```
RadioPanel m_radioPanel
```

---

### m\_currentDecoration

```
DecorationEnum m_currentDecoration
```

---

### m\_plainButton

```
JButton m_plainButton
```

---

### m\_wireframeButton

```
JButton m_wireframeButton
```

---

### m\_textLabelsButton

```
JButton m_textLabelsButton
```

---

### m\_patternsButton

```
JButton m_patternsButton
```

---

### m\_halftoningButton

```
JButton m_halftoningButton
```

---

### m\_patternsMenu

```
JComboBox m_patternsMenu
```

---

### m\_halftoningMenu

```
JComboBox m_halftoningMenu
```

---

### m\_bendMenu

```
JComboBox m_bendMenu
```

---

### m\_extraLinesCheckBox

```
JCheckBox m_extraLinesCheckBox
```

**Class org.proteinshader.gui.components.controlpanel.ModifierPanel extends JPanel implements Serializable**

**Serialized Fields**

### m\_factory

```
ModifierPanelListenerFactory m_factory
```

---

### m\_mediator

```
Mediator m_mediator
```

---

### m\_dialogOwner

```
Frame m_dialogOwner
```

---

### m\_subpanelMenu

```
JComboBox m_subpanelMenu
```

---

### m\_defaultSubpanel

```
JPanel m_defaultSubpanel
```

---

### m\_centerPanel

```
JPanel m_centerPanel
```

---

### m\_atomRadioPanel

```
RadioPanel m_atomRadioPanel
```

---

### m\_cartoonRadioPanel

```
RadioPanel m_cartoonRadioPanel
```

---

### m\_cartoonColorPanel

```
CartoonColorPanel m_cartoonColorPanel
```

---

### m\_cartoonVisibilityPanel

```
CartoonVisibilityPanel m_cartoonVisibilityPanel
```

---

### m\_cartoonSideChainsPanel

```
CartoonSideChainsPanel m_cartoonSideChainsPanel
```

---

### m\_decorationsPanel

```
DecorationsPanel m_decorationsPanel
```

---

### m\_atomColorPanel

```
AtomColorPanel m_atomColorPanel
```

---

### m\_atomVisibilityPanel

```
AtomVisibilityPanel m_atomVisibilityPanel
```

---

### m\_atomScalePanel

```
AtomScalePanel m_atomScalePanel
```

---

### m\_motionPanel

```
MotionPanel m_motionPanel
```

---

### m\_antialiasingPanel

```
AntialiasingPanel m_antialiasingPanel
```

---

### m\_optimizePanel

```
OptimizePanel m_optimizePanel
```

**Class org.proteinshader.gui.components.controlpanel.MotionPanel extends JPanel implements Serializable**

**Serialized Fields**

### m\_factory

```
MotionPanelListenerFactory m_factory
```

---

### m\_numberFormatter

```
NumberFormat m_numberFormatter
```

---

### m\_mediator

```
Mediator m_mediator
```

---

### m\_dialogOwner

```
Frame m_dialogOwner
```

---

### m\_xAxisSlider

```
JSlider m_xAxisSlider
```

---

### m\_yAxisSlider

```
JSlider m_yAxisSlider
```

---

### m\_startButton

```
JButton m_startButton
```

---

### m\_stopButton

```
JButton m_stopButton
```

---

### m\_fpsTextField

```
JTextField m_fpsTextField
```

---

### m\_xAxisTextField

```
JFormattedTextField m_xAxisTextField
```

---

### m\_yAxisTextField

```
JFormattedTextField m_yAxisTextField
```

---

### m\_animationIsRunning

```
boolean m_animationIsRunning
```

---

### m\_debug

```
boolean m_debug
```

**Class org.proteinshader.gui.components.controlpanel.OptimizePanel extends JPanel implements Serializable**

**Serialized Fields**

### m\_factory

```
OptimizePanelListenerFactory m_factory
```

---

### m\_mediator

```
Mediator m_mediator
```

---

### m\_dialogOwner

```
Frame m_dialogOwner
```

---

### m\_autoTilingButton

```
JCheckBox m_autoTilingButton
```

---

### m\_centerPanel

```
JPanel m_centerPanel
```

---

### m\_spheresButton

```
JRadioButton m_spheresButton
```

---

### m\_cylindersButton

```
JRadioButton m_cylindersButton
```

---

### m\_cylinderCapsButton

```
JRadioButton m_cylinderCapsButton
```

---

### m\_spheresMenu

```
JComboBox m_spheresMenu
```

---

### m\_cylindersMenu

```
JComboBox m_cylindersMenu
```

---

### m\_cylinderCapsMenu

```
JComboBox m_cylinderCapsMenu
```

---

### m\_sphereTilingLabel

```
JLabel m_sphereTilingLabel
```

---

### m\_cylinderTilingLabel

```
JLabel m_cylinderTilingLabel
```

---

### m\_cylinderCapsTilingLabel

```
JLabel m_cylinderCapsTilingLabel
```

---

### m\_tilingSpinner

```
JSpinner m_tilingSpinner
```

---

### m\_applyButton

```
JButton m_applyButton
```

---

### m\_defaultButton

```
JButton m_defaultButton
```

---

### m\_debug

```
boolean m_debug
```

**Class org.proteinshader.gui.components.controlpanel.RadioPanel extends JPanel implements Serializable**

**Serialized Fields**

### m\_factory

```
RadioPanelListenerFactory m_factory
```

---

### m\_visitor

```
ModifierVisitor m_visitor
```

---

### m\_mediator

```
Mediator m_mediator
```

---

### m\_dialogOwner

```
Frame m_dialogOwner
```

---

### m\_type

```
RadioPanel.Type m_type
```

---

### m\_upperPanel

```
JPanel m_upperPanel
```

---

### m\_lowerPanel

```
JPanel m_lowerPanel
```

---

### m\_selectedButton

```
JRadioButton m_selectedButton
```

---

### m\_helicesButton

```
JRadioButton m_helicesButton
```

---

### m\_strandsButton

```
JRadioButton m_strandsButton
```

---

### m\_loopsButton

```
JRadioButton m_loopsButton
```

---

### m\_globalButton

```
JRadioButton m_globalButton
```

---

### m\_selectedMenu

```
JComboBox m_selectedMenu
```

---

### m\_helicesMenu

```
JComboBox m_helicesMenu
```

---

### m\_strandsMenu

```
JComboBox m_strandsMenu
```

---

### m\_loopsMenu

```
JComboBox m_loopsMenu
```

---

### m\_globalMenu

```
JComboBox m_globalMenu
```

---

### m\_applyToMenu

```
JComboBox m_applyToMenu
```

---

### m\_activeRadioButton

```
RadioButtonEnum m_activeRadioButton
```

**Class org.proteinshader.gui.components.controlpanel.SelectorPanel extends JPanel implements Serializable**

**Serialized Fields**

### m\_factory

```
SelectorPanelListenerFactory m_factory
```

---

### m\_mediator

```
Mediator m_mediator
```

---

### m\_residuesLabel

```
JLabel m_residuesLabel
```

---

### m\_atomsLabel

```
JLabel m_atomsLabel
```

---

### m\_residueIDLabel

```
JLabel m_residueIDLabel
```

---

### m\_modelMenu

```
JComboBox m_modelMenu
```

---

### m\_chainMenu

```
JComboBox m_chainMenu
```

---

### m\_residueMenu

```
JComboBox m_residueMenu
```

---

### m\_residueListModel

```
DefaultListModel m_residueListModel
```

---

### m\_atomListModel

```
DefaultListModel m_atomListModel
```

---

### m\_residueList

```
JList m_residueList
```

---

### m\_atomList

```
JList m_atomList
```

---

### m\_atomListDialog

```
JDialog m_atomListDialog
```

---

### m\_dialogOwner

```
Frame m_dialogOwner
```

---

### m\_atomsButton

```
JButton m_atomsButton
```

---

### m\_atomCloseButton

```
JButton m_atomCloseButton
```

---

### m\_debug

```
boolean m_debug
```

**Class org.proteinshader.gui.components.controlpanel.VisibilityPanel extends JPanel implements Serializable**

**Serialized Fields**

### m\_factory

```
VisibilityPanelListenerFactory m_factory
```

---

### m\_mediator

```
Mediator m_mediator
```

---

### m\_dialogOwner

```
Frame m_dialogOwner
```

---

### m\_radioPanel

```
RadioPanel m_radioPanel
```

---

### m\_percentField

```
JFormattedTextField m_percentField
```

---

### m\_alphaSlider

```
JSlider m_alphaSlider
```

---

### m\_opaqueButton

```
JButton m_opaqueButton
```

---

### m\_invisibleButton

```
JButton m_invisibleButton
```

---

### m\_translucentButton

```
JButton m_translucentButton
```

---

**Package** **org.proteinshader.gui.components.menubar**

**Class org.proteinshader.gui.components.menubar.AboutBox extends JDialog implements Serializable**

**Serialized Fields**

### m\_listenerFactory

```
AboutBoxListenerFactory m_listenerFactory
```

---

### m\_closeButton

```
JButton m_closeButton
```

---

### m\_webSiteButton

```
JButton m_webSiteButton
```

**Class org.proteinshader.gui.components.menubar.BackgroundMenu extends JMenu implements Serializable**

**Serialized Fields**

### m\_factory

```
BackgroundMenuListenerFactory m_factory
```

---

### m\_chooser

```
JColorChooser m_chooser
```

---

### m\_chooserDialog

```
JDialog m_chooserDialog
```

---

### m\_dialogOwner

```
Frame m_dialogOwner
```

---

### m\_blackMenuItem

```
JMenuItem m_blackMenuItem
```

---

### m\_grayMenuItem

```
JMenuItem m_grayMenuItem
```

---

### m\_lightGrayMenuItem

```
JMenuItem m_lightGrayMenuItem
```

---

### m\_whiteMenuItem

```
JMenuItem m_whiteMenuItem
```

---

### m\_customMenuItem

```
JMenuItem m_customMenuItem
```

**Class org.proteinshader.gui.components.menubar.FileMenu extends JMenu implements Serializable**

**Serialized Fields**

### m\_factory

```
FileMenuListenerFactory m_factory
```

---

### m\_openMenuItem

```
JMenuItem m_openMenuItem
```

---

### m\_jpgFileItem

```
JMenuItem m_jpgFileItem
```

---

### m\_pngFileItem

```
JMenuItem m_pngFileItem
```

---

### m\_gifFileItem

```
JMenuItem m_gifFileItem
```

---

### m\_invisibleCanvasPngFileItem

```
JMenuItem m_invisibleCanvasPngFileItem
```

---

### m\_quitMenuItem

```
JMenuItem m_quitMenuItem
```

**Class org.proteinshader.gui.components.menubar.HelpMenu extends JMenu implements Serializable**

**Serialized Fields**

### m\_factory

```
HelpMenuListenerFactory m_factory
```

---

### m\_mediator

```
Mediator m_mediator
```

---

### m\_dialogOwner

```
Frame m_dialogOwner
```

---

### m\_aboutMenuItem

```
JMenuItem m_aboutMenuItem
```

---

### m\_localHelpMenuItem

```
JMenuItem m_localHelpMenuItem
```

---

### m\_sourceForgeMenuItem

```
JMenuItem m_sourceForgeMenuItem
```

---

### m\_graphicsCardMenuItem

```
JMenuItem m_graphicsCardMenuItem
```

**Class org.proteinshader.gui.components.menubar.MainMenuBar extends JMenuBar implements Serializable**

**Serialized Fields**

### m\_visibilityMenu

```
VisibilityMenu m_visibilityMenu
```

---

### m\_toolsMenu

```
ToolsMenu m_toolsMenu
```

---

### m\_styleMenu

```
StyleMenu m_styleMenu
```

---

### m\_helpMenu

```
HelpMenu m_helpMenu
```

**Class org.proteinshader.gui.components.menubar.OrientationMenu extends JMenu implements Serializable**

**Serialized Fields**

### m\_factory

```
OrientationMenuListenerFactory m_factory
```

**Class org.proteinshader.gui.components.menubar.StyleMenu extends JMenu implements Serializable**

**Serialized Fields**

### m\_factory

```
StyleMenuListenerFactory m_factory
```

---

### m\_mediator

```
Mediator m_mediator
```

---

### m\_defaultRadioButton

```
JRadioButtonMenuItem m_defaultRadioButton
```

**Class org.proteinshader.gui.components.menubar.ToolsMenu extends JMenu implements Serializable**

**Serialized Fields**

### m\_factory

```
ToolsMenuListenerFactory m_factory
```

---

### m\_controlPanelCheckBox

```
JCheckBoxMenuItem m_controlPanelCheckBox
```

**Class org.proteinshader.gui.components.menubar.VisibilityMenu extends JMenu implements Serializable**

**Serialized Fields**

### m\_factory

```
VisibilityMenuListenerFactory m_factory
```

---

### m\_aminoAcidsCheckBox

```
JCheckBoxMenuItem m_aminoAcidsCheckBox
```

---

### m\_heterogensCheckBox

```
JCheckBoxMenuItem m_heterogensCheckBox
```

---

### m\_watersCheckBox

```
JCheckBoxMenuItem m_watersCheckBox
```

---

**Package** **org.proteinshader.gui.exceptions**

**Class org.proteinshader.gui.exceptions.ScreenShotException extends Exception implements Serializable**

**Serialized Fields**

### m\_format

```
ImageFormatEnum m_format
```

---

### m\_file

```
File m_file
```

---

### m\_lowerLevelMessage

```
String m_lowerLevelMessage
```

---

**Package** **org.proteinshader.gui.utils**

**Class org.proteinshader.gui.utils.CenteredListCellRenderer extends DefaultListCellRenderer implements Serializable**

**Class org.proteinshader.gui.utils.ImageFileChooser extends JFileChooser implements Serializable**

**Serialized Fields**

### m\_format

```
ImageFormatEnum m_format
```

**Class org.proteinshader.gui.utils.PaddedListCellRenderer extends DefaultListCellRenderer implements Serializable**

**Class org.proteinshader.gui.utils.SeparatorListCellRenderer extends DefaultListCellRenderer implements Serializable**

**Serialized Fields**

### m\_separators

```
String[] m_separators
```

---

### m\_lineColor

```
Color m_lineColor
```

---

**Package** **org.proteinshader.structure.exceptions**

**Class org.proteinshader.structure.exceptions.BondLengthException extends Exception implements Serializable**

**Serialized Fields**

### m\_calculatedLength

```
double m_calculatedLength
```

---

### m\_expectedLength

```
double m_expectedLength
```

---

### m\_toleranceFactor

```
double m_toleranceFactor
```

---

### m\_srcAtomType

```
AtomEnum m_srcAtomType
```

---

### m\_dstAtomType

```
AtomEnum m_dstAtomType
```

---

### m\_srcAtomSerialNo

```
int m_srcAtomSerialNo
```

---

### m\_dstAtomSerialNo

```
int m_dstAtomSerialNo
```

**Class org.proteinshader.structure.exceptions.ColorOutOfRangeException extends Exception implements Serializable**

**Class org.proteinshader.structure.exceptions.InvalidIDException extends Exception implements Serializable**

**Class org.proteinshader.structure.exceptions.InvalidRegionException extends Exception implements Serializable**

**Class org.proteinshader.structure.exceptions.MissingAATypeException extends Exception implements Serializable**

**Class org.proteinshader.structure.exceptions.MissingAtomTypeException extends Exception implements Serializable**

**Class org.proteinshader.structure.exceptions.MissingHetNameException extends Exception implements Serializable**

**Class org.proteinshader.structure.exceptions.SegmentException extends Exception implements Serializable**

---

**Package** **org.proteinshader.structure.io.exceptions**

**Class org.proteinshader.structure.io.exceptions.PDBFieldExtractorException extends Exception implements Serializable**

**Class org.proteinshader.structure.io.exceptions.PDBLineParserException extends Exception implements Serializable**

**Class org.proteinshader.structure.io.exceptions.StructureReaderException extends Exception implements Serializable**

---

**Package** **org.proteinshader.structure.visitor.exceptions**

**Class org.proteinshader.structure.visitor.exceptions.VisitorException extends Exception implements Serializable**

**Class org.proteinshader.structure.visitor.exceptions.WriterVisitorException extends VisitorException implements Serializable**

---


|  |  |  |
| --- | --- | --- |
| **Overview** \| Package \| Class \| Use \| **Tree** \| **Deprecated** \| **Index** \| **Help** | |  |
| PREV   NEXT | **FRAMES**    **NO FRAMES**     **All Classes** |  |


---

# *Copyright © 2007-2008*
